# The effect of nurses’ preparedness and nurse practitioner status on triage call management in primary care: A secondary analysis of cross-sectional data from the ESTEEM trial

**DOI:** 10.1016/j.ijnurstu.2016.02.001

**Published:** 2016-06

**Authors:** Anna Varley, Fiona C. Warren, Suzanne H. Richards, Raff Calitri, Katherine Chaplin, Emily Fletcher, Tim A. Holt, Valerie Lattimer, Jamie Murdoch, David A. Richards, John Campbell

**Affiliations:** a University of East Anglia, United Kingdom; b University of Exeter, United Kingdom; c University of Bristol, United Kingdom; d University of Oxford, United Kingdom

**Keywords:** Clinical experience, Confidence, Implementation, Nurse telephone triage, Primary care, Training

## Abstract

**Background:**

Nurse-led telephone triage is increasingly used to manage demand for general practitioner consultations in UK general practice. Previous studies are equivocal about the relationship between clinical experience and the call outcomes of nurse triage. Most research is limited to investigating nurse telephone triage in out-of-hours settings.

**Objective:**

To investigate whether the professional characteristics of primary care nurses undertaking computer decision supported software telephone triage are related to call disposition.

**Design:**

Questionnaire survey of nurses delivering the nurse intervention arm of the ESTEEM trial, to capture role type (practice nurse or nurse practitioner), prescriber status, number of years’ nursing experience, graduate status, previous experience of triage, and perceived preparedness for triage.

Our main outcome was the proportion of triaged patients recommended for follow-up within the practice (call disposition), including all contact types (face-to-face, telephone or home visit), by a general practitioner or nurse.

**Settings:**

15 general practices and 7012 patients receiving the nurse triage intervention in four regions of the UK.

**Participants:**

45 nurse practitioners and practice nurse trained in the use of clinical decision support software.

**Methods:**

We investigated the associations between nursing characteristics and triage call disposition for patient ‘same-day’ appointment requests in general practice using multivariable logistic regression modelling.

**Results:**

Valid responses from 35 nurses (78%) from 14 practices: 31/35 (89%) had ≥10 years’ experience with 24/35 (69%) having ≥20 years. Most patient contacts (3842/4605; 86%) were recommended for follow-up within the practice. Nurse practitioners were less likely to recommend patients for follow-up odds ratio 0.19, 95% confidence interval 0.07; 0.49 than practice nurses. Nurses who reported that their previous experience had prepared them less well for triage were more likely to recommend patients for follow-up (OR 3.17, 95% CI 1.18–5.55).

**Conclusion:**

Nurse characteristics were associated with disposition of triage calls to within practice follow-up. Nurse practitioners or those who reported feeling ‘more prepared’ for the role were more likely to manage the call definitively. Practices considering nurse triage should ensure that nurses transitioning into new roles feel adequately prepared. While standardised training is necessary, it may not be sufficient to ensure successful implementation.

**What is already known about the topic?**

• Previous studies indicate a lack of consistency between decisions made by healthcare professionals of different clinical backgrounds in a range of primary and emergency care settings.
• There is evidence to indicate that length of clinical experience and the characteristics of triaging nurses may impact triage call disposition in emergency and out-of-hours care settings.
• Little is known about factors affecting nurse triage in routine, primary care services.

**What this paper adds**

• We investigated the associations between nursing characteristics (e.g. level of experience, qualifications) and triage call disposition for patient ‘same-day’ appointment requests in general practice.
• We found that nurse practitioners were more likely to definitively manage the patient within a triage consultation than practice nurses, while nurses who reported lower levels of ‘preparedness’ for triage were more likely to recommend that the patient received a follow-up consultation.
• Practices considering implementing nurse triage should ensure that nurses transitioning into new roles feel adequately prepared, as while standardised training is necessary, it may not be sufficient to ensure successful implementation.

## 1 Introduction

Telephone triage by nurses and physicians has become increasingly popular both in the UK and internationally over the last decade (Bunn et al., 2005, Mohammed et al., 2012, Venning et al., 2000). In the UK the National Health Service provides a free universal healthcare system covering both primary and secondary care. General practitioner (GP) surgeries are the first point of the contact for the majority of non-emergency health conditions in primary care. General practices are increasingly struggling to meet patient demand (Drayan et al., 2015). The use of telephone triage is one strategy being employed to manage the increasing workloads (Salisbury et al., 2007). Nurses have been shown to provide a safe and effective triage service in a variety of settings including primary care where it can be an effective way to manage GP workload on the day of contact (Campbell et al., 2014, Huibers et al., 2012, Kinnersley et al., 2000, Richards et al., 2004) and out-of-hours primary medical care services (Lattimer et al., 1998). In primary care settings, patients have been found to be at least as satisfied, if not more satisfied, with face-to-face consultations with nurse practitioners compared with doctors (Horrocks et al., 2002, Laurant et al., 2005). Research has also found that patients broadly accept nursing roles extending to tasks traditionally undertaken by doctors (Caldow et al., 2007).

Despite the extension of nursing roles, questions remain over the quality of nurse triage decision making. Previous studies indicate a lack of consistency between decisions made by healthcare professionals of different clinical backgrounds in a range of primary and emergency care settings (Durand et al., 2011, O’Donnell, 2000). Research focussing on nurses’ decision making has similarly discovered variation in outcomes in different settings. In the UK, nurses using standardised patient scenarios to test their telephone triage decision making for the ‘NHS Direct’, a 24 h health telephone advice service, exhibited a lack of consistency between decisions made (O’Cathain et al., 2003). This concurs with Canadian research for an emergency triage service examining ‘real-world’ calls (Leprohon and Patel, 1995) and data from an out-of-hours primary medical care cooperative in the Netherlands that observed considerable variability between nurses in the proportion of calls resolved by the triage call alone rather than resulting in onward referral (Moll van Charante et al., 2006).

Nurse characteristics, such as length of experience and level of qualification, may also influence triage outcomes. A study of 60,794 calls managed by 296 nurses in NHS Direct reported a positive relationship with years of nursing experience and call disposal patterns (O’Cathain et al., 2004). Nurses with less than 10 years’ clinical experience were less likely to dispose calls to self-care than nurses with more than 20 years’ experience. This is consistent with other research observing that nurses with more experience demonstrated improved accuracy/correctness of triage outcome chosen (Cioffi, 1998, Leprohon and Patel, 1995), lower levels of data collection during triage assessments, and more judgements (inferences) made based on prior experience (Cioffi, 1998). Positive correlations have also been observed between nurses with a post-secondary level qualification (compared with nurses with no additional training) and achieving the ‘expected triage decision’ as opposed to ‘overtriage’ or ‘undertriage’ in emergency care settings (Considine et al., 2001).

Overall, there is evidence to indicate that length of clinical experience and the characteristics of triaging nurses may have an impact on triage call disposition in emergency and out-of-hour care settings, but little is known about factors affecting nurse triage in routine, primary care services. We recently conducted a large-scale multicentre cluster randomised controlled trial (RCT) (Campbell et al., 2013, Campbell et al., 2014), which compared GP-led and nurse-led triage with usual care for the management of requests for a same-day GP consultation. Nurse-led triage resulted in an increase in the number of subsequent primary care contacts (a composite measure comprised of face to face, home visits and contacts by telephone with nurses and GPs) during the 28-day follow-up period, compared with usual care, with a 48% increase observed (rate ratio (RR) 1.48, 95% confidence interval (CI) 1.44–1.52). However, in the nurse-led triage arm, a 20% reduction in GP face-to-face contacts was observed compared with usual care, across the 28-day follow-up period (RR 0.80, 95% CI 0.71–0.90) (Campbell et al., 2015). We conducted a post hoc analysis of the ESTEEM data regarding the outcome of nurse triage telephone consultations to determine whether nurses’ background, prior clinical experience, and perceived preparedness for the role of triage nurse, were associated with call disposition.

## 2 Methods

ESTEEM was a cluster RCT of 21,000 patients requesting same day appointments in 42 general practices across four regions of England. Patients were recruited between May 2011 and December 2012 across 42 practices, of which 15 were nurse triage practices, recruiting 7012 patients. A detailed description of the ESTEEEM trial methodology, including details of the sample size calculation, can be found elsewhere (Campbell et al., 2013, Campbell et al., 2014, Campbell et al., 2015).

### 2.1 Population

As part of the ESTEEM trial, 15 practices were randomised to the nurse triage arm. Practices were recruited from four regions of England, UK (Devon, Bristol/Somerset, Warwickshire/Coventry, Norfolk/Suffolk). Although only qualified nurses were eligible to deliver telephone triage, no other level of qualification was stipulated and thus practices selected nurses with varying professional characteristics and levels of prior experience to undertake telephone triage. The nurses who took part were a mixture of those who identified themselves as practice nurses and those who identified themselves as nurse practitioners. Although some characteristics of nurse practitioners are country-specific, it is widely accepted that they are nurses with a high level of autonomous working and able to make independent diagnoses and treatment decisions (RCN, 2012, Schober and Affara, 2006). The nurse practitioner role is characterised by a more complex level of skills and abilities, resulting in a higher level of experience and distinct role to that of a practice nurse. The nurse practitioner will have undertaken an extended amount of training often to masters level and very often also have the ability to prescribe. There is currently no national data available that identifies the number/proportions of practice nurses who are categorised as nurse practitioners or nurse prescribers. However, data from the NHS Health and Social Care Information Centre reports that in 2014 ‘advanced nurses’ accounted for 23% of practice nurses working in general practice (3507/15,062) and nurses working in extended roles including prescribers accounted for 20% (2960/15,062). An increase in the number of nurses prescribing in general practice has been identified, however the numbers of nurse prescriptions remain very low when compared with prescriptions by physicians. In 2010, only 1.5% of the total items prescribed in primary care were prescribed by nurses (Drennan et al., 2014).

### 2.2 Intervention: triage training and implementation

All nurses undertook a bespoke training programme to enable them to use clinical decision support software (CDSS) provided by Plain Healthcare within telephone triage consultations. All nurses to be involved in triage duties were required to receive this standardised training package in the use of CDSS and telephone consultations skills. Following initial face to face training, nurses received one-to-one remote training whilst they practised using the CDSS on simulated patient scenarios. The nurses’ use of the system was then assessed by Plain Healthcare trainers, with all nurses needing to demonstrate proficiency in its use before delivering telephone triage within the trial. Following this, there was a 1 month period during which nurses practised using CDSS in simulated patient scenarios during their daily work prior to beginning trial procedures.

It is important to note that the CDSS is designed to support the nurse's clinical decision making; although the nurses were required to open the software for each consultation there was no requirement in the trial that the recommended advice the CDSS generated had to be followed. The intention of using the CDSS was therefore to support nurses in their decision-making and to provide them with flexibility to use their own knowledge and experience to decide the outcome of the triage call.

Although the core triage processes were specified, practices had some areas of flexibility in implementing the nurse telephone triage intervention. Two alternative staffing models emerged regarding which nurses delivered the triage: either one nurse at a time conducted a triage session (on a rotating basis) or a number of nurses shared triaging duties during the day. Practices were asked to triage all consecutive eligible patients during opening hours to ensure the least disruption and shortest time of trial data collection possible. In the event that limited staff resources prevented triage of all eligible patients each day, practices were permitted to operate triage within specific agreed sessions (‘Research Windows’), amounting to no less than 50% of the week and encompassing all five working days and both mornings and afternoons. The length of time practices undertook nurse telephone triage and trial data collection ranged between 2 and 15 weeks. The smallest number of patients with call disposal data available that were triaged by an individual nurse was 18; the largest number was 293.

## 3 Measures

### 3.1 Patient data extracted from clinician form (primary outcome modelled)

Our primary outcome was recommended call disposition, dichotomised into a binary variable indicating whether or not follow-up was required. We collected data on recommended patient follow-up within the practice, including all contact types (face-to-face, telephone or home visit, by a GP or nurse, with no restrictions on the recommended timescale for follow-up). We derived patient-level data from a short data collection form (‘Clinician Form’) completed for each patient within the trial by the triaging nurse. Nurses completed the form during the initial consultation following a patient's same-day appointment request. The initials of each nurse were recorded so that data could be linked to the triaging nurse's demographic and professional data.

### 3.2 Potential explanatory variables from the clinician form

Patient-level covariates included (i) gender (female as reference); (ii) age divided into six categories: 0–4 years; 5–11 years; 16–24 years; 25–59 years (reference category); 60–74 years; 75 years and older; and (iii) patient-level deprivation divided into quintiles based on rank (reference: least deprived quintile). Patients’ deprivation status was based on IMD 2010 score (UK Government Indices of Multiple Deprivation 2010; URL: www.gov.uk/government/publications/english-indices-of-deprivation-2010) and rank of patients’ residential lower super output area (LSOA), obtained by mapping residential postcode to the relevant LSOA.

### 3.3 Nurse survey

We collected nurse demographic and professional characteristics via a self-reported postal questionnaire. We sent the questionnaire invitations directly to all 45 nurses who conducted telephone triage in the trial asking them to take part. Demographic characteristics collected included age (18–24, 25–34,/35–44,/45–54, and 55–64 years), gender, and ethnicity. Professional characteristics included self-reported role title (nurse practitioner, practice nurse); whether the individual was a nurse prescriber (no/yes); the academic level of their professional qualifications (diploma, undergraduate, postgraduate); the number of settings in which they had gained experience in primary and secondary care (one, two, or three or more); and the total number of years’ experience in primary care and other settings (≤10 years, 10–19 years, ≥20 years) since registration. We also asked nurses whether they had prior experience of triage and CDSS, and how well they felt their previous experience prior to the trial had prepared them for triaging (very well, moderately well, not well). We worked with practice managers to remind non-respondents on one occasion only of the opportunity to complete the survey, after which no further reminders were issued.

### 3.4 Practice level data collection

Practice-level covariates included: (i) location; (ii) practice list size: large (more than 8000 registered patients; reference); medium (3500–8000 registered patients), small (less than 3500 registered patients); and (iii) practice-level deprivation (obtained from Public Health England National General Practice Profiles (APHO, 2013); non-deprived: average/below average deprivation for England (reference); deprived: above average deprivation for England).

## 4 Data analysis

Our primary outcome was recommended call disposition, dichotomised into a binary variable indicating whether or not follow-up within practice was required. All statistical analyses comprised logistic hierarchical models with individual patient observations nested within nurse, and nurses nested within practice, thus nurse and practice were included as random effects. All analyses included fixed effect practice and patient-level covariates. Practice-level covariates comprised location, practice list size, and practice-level deprivation. Patient-level covariates comprised age, gender, and patient-level deprivation (Campbell et al., 2015). We only included data if the contact was a nurse telephone contact on the day of the patient's initial telephone call to the practice requesting a same-day GP consultation. The seven nurse characteristics investigated as fixed effects within the inferential analyses were: role; prescriber status; number of years nursing experience; nursing graduate; number of settings of nurse experience (dichotomised as one or two versus three or more); previous experience of triage; and whether the nurse felt that their previous experience had prepared them well for triage (perceived preparedness; dichotomised as very well versus moderately well or not well). We developed a series of multivariable regression models to determine those nurse characteristics that were significantly associated with follow-up within practice, individually and in combination with other significant nurse characteristics (with adjustment for patient- and practice-level covariates). We investigated each individual nurse characteristic for association with follow-up within practice. A further model included all nurse characteristics. Any nurse characteristic that was significant either individually or within the model including all nurse characteristics was taken forward to a further model. We then removed any non-significant nurse characteristics from this model; we then added characteristics that were previously non-significant to the model individually to determine whether their addition improved model fit.

We derived marginal probabilities of follow-up within practice (derived from the fixed-effect portion of the hierarchical model) for significant characteristics, based on the assumption that all observations in the dataset took the same value for each characteristic in turn (for example, assuming first that all nurses were practice nurses, and then that all nurses were nurse practitioners) while all other covariates in the model retained their observed values. We set the significance threshold at 0.1 in order to determine whether to consider a nurse characteristic in further models, and 0.05 for inclusion into the final model. All logistic regression models were conducted using STATA v. 12.

## 5 Results

### 5.1 Nurse participants

Of the 45 nurses who conducted telephone triage as part of the trial, 6 (13%) were unable to return the questionnaire (5 had left the practice and 1 was on leave). Of those able to reply, 35/39 (90%) returned a completed questionnaire. Replies were received from 14/15 (93%) practices in which nurse triage was undertaken.

The self-reported characteristics of nurses are presented in Table 1. All eight of the nurse practitioners were prescribers and an additional three practice nurses (3/27; 11%) could also prescribe. Hence, there was a high degree of correlation between nurse role (nurse practitioner versus practice nurse) and prescriber status. Also, all eight nurse practitioners had 20 or more years’ experience, compared with 16/27 (59%) practice nurses. A greater proportion of nurse practitioners (5/7; 71%) reported that they were well-prepared for triage (versus moderately/not well prepared) compared with practice nurses (13/26; 50%). Furthermore, 12/15 (80%) nurses with triage experience stated that they were well-prepared for triage compared with only 6/18 (33%) of those without triage experience.

### 5.2 Patient sample

**Fig. 1:**
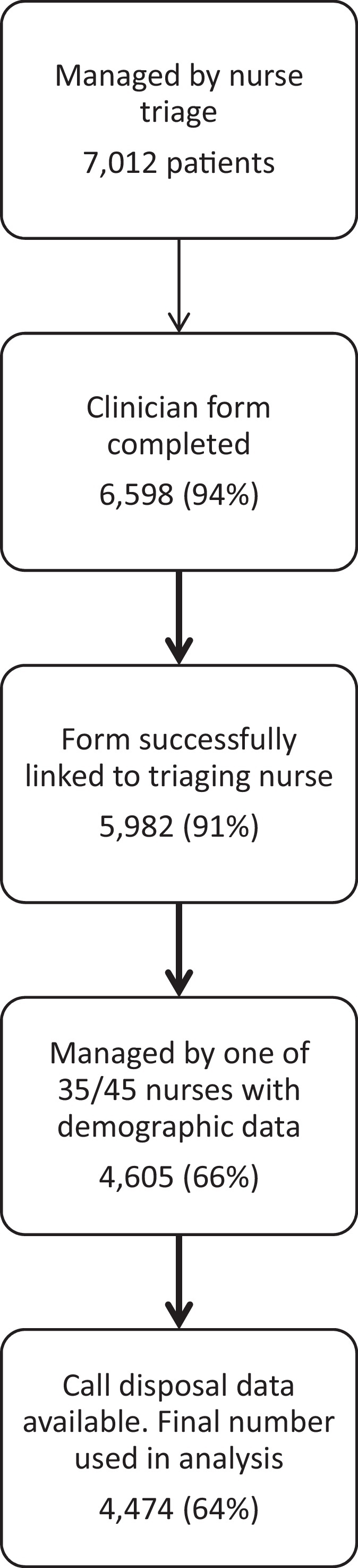
Number of patients contributing data to the modelling.

Fig. 1 summarises the selection of patient data included in our sample. Detailed data on the patient sample is reported elsewhere (Campbell et al., 2015).

### 5.3 Call disposition

Of 4474 patients who received nurse telephone triage on the day of the index call, 3842 (86%) were recommended for follow-up within practice. Two nurses referred all of their triaged patients for within-practice follow-up (18 and 40 patients respectively); the smallest proportion of patients referred for within-practice follow-up by a nurse was 66/104 (63%). The main trial did not collect data on the clinical content of the calls and therefore was unable to judge as to whether the decision to refer for within-practice follow-up was the most appropriate follow-up option.

Our multi-level logistic regression analyses indicated very little between-practice variation in call disposition once between-nurse variation was accounted for; hence, we have reported only models with two levels, individual patients nested within nurse.

In those models including only one nurse characteristic, nurse role, prescriber status, previous triage experience, number of settings of nurse experience, and perceived preparedness, were significantly associated with the outcome of call disposition (Table 2), and thus selected for further analysis. Including all seven nurse characteristics in one model, only nurse practitioner status and perceived preparedness were significantly associated with follow-up (Table 2). Including nurse role, prescriber status, previous triage experience, number of settings of nurse experience, and level of preparedness into one model, only nurse practitioner status, prescriber status and perceived preparedness remained significant (data not shown). On adding other nurse characteristics individually to the model including nurse practitioner status, prescriber status, and perceived preparedness, no further nurse characteristics were significantly associated with follow-up (data not shown). Based on the final model, including nurse practitioner status, prescriber status, and perceived preparedness (Table 3), the marginal probability for a patient being followed-up in practice was 0.90 with a 95% CI of 0.88–0.93 for practice nurses (Table 3); for nurse practitioners the probability was 0.66 (95% CI 0.51–0.81). With regard to perceived preparedness, those nurses who considered themselves to be well-prepared had a marginal probability for within-practice follow-up of 0.57 (95% CI 0.40–0.75) and those who said they were not well prepared or moderately well prepared had a marginal probability of 0.79 (0.74–0.85).

## 6 Discussion

We found significant correlation between nursing role and preparedness for triage and the likelihood that a nurse would recommend a patient for within-practice follow-up. Nurse practitioners had a lower probability of disposing patients to a further within-practice follow-up than those who described themselves as practice nurses. Nurse practitioners are also likely to be more confident nurses in that they have chosen to undertake further training to take on this role. This may also extend to feeling more confident and prepared to undertake triage. Our results support current research on the benefit of using nurse practitioners in primary care (Horrocks et al., 2002, Kinnersley et al., 2000).

A significant association was found between nurses having a perception of being ‘very prepared’ as opposed to ‘moderately well/not well prepared’. Those nurses who felt less prepared had a significantly higher rate of call disposal to within practice follow up. Although the nurses in the trial had various levels of previous experience and expertise; they all received the same amount of training to carry out telephone triage and passed a proficiency test to use the CDSS. The results from the series of multivariable models demonstrated that perceived preparedness was more strongly associated with disposal of call to within practice follow-up than previous triage experience; this is notable in the light of the fact that these factors were highly collinear. It is therefore possible that perceived preparedness to perform triage is more strongly associated with confidence to manage patients definitively by telephone (without recall for further consultation) than actual previous experience of triage. Perceived preparedness can be viewed as a proxy for confidence and this was a factor frequently mentioned by participants in a qualitative study of nurses working in NHS Direct (O’Cathain et al., 2004). The authors found some indication of a relationship between confidence and length of experience however, some very experienced nurses were cautious about the decisions they made. Therefore, confidence alone may be a factor that influences decision making. In the ESTEEM trial process evaluation (Campbell et al., 2015) it was noted that some nurses were frequently nervous about conducting triage, which sometimes lay beyond the comfort zone of their previous experience and training. This could explain why nurses who felt more prepared and therefore more confident about carrying out triage disposed fewer patients for within practice follow-up than more experienced but less confident nurses.

Contrary to previous research, we found no evidence that length of clinical experience (20 years or more versus 10–20 years and less than 10 years) was associated with calls disposed to within-practice follow-up, with adjustment for other nurse characteristics. This is consistent with the findings of several other studies (Considine et al., 2001, Considine et al., 2007, Moll van Charante et al., 2006), but does not concur with the work in NHS Direct (O’Cathain et al., 2004). This could be for a number of reasons, not least of all that the settings of NHS Direct and General Practice are very different environments in which to carry out telephone triage. The volume of calls received by NHS Direct compared to general practice is vastly higher. Another factor may be that the nurses in our study had not been triaging for very long in their current role (they only had a 3–4 week run in period) whereas the NHS Direct nurses had been triaging for much longer (60% had been working in NHS Direct for over 6 months) and this could explain why these nurses referred fewer patients for follow up. A sub-study of ESTEEM which audio-recorded nurse–patient interactions and video recorded their use of the CDDS during triage calls found that despite their training nurses had some difficulties in using the software and orientating to patient's needs (Murdoch et al., 2015a). Such difficulties may have undermined nurse's confidence in determining triage outcome regardless of clinical experience. In contrast, NHS Direct nurses had clearly been using the CDSS for much longer and therefore may explain why years of experience was shown to be beneficial in O’Cathain's study. Added to this a number of nurses who were interviewed as part of the ESTEEM process evaluation reflected that the CDSS software was not suitable for use in a primary care setting (Murdoch et al., 2015b). There are therefore a combination of factors which might have all contributed to the nurses referring a higher proportion of patients for follow-up within the practice.

Prescriber status was highly correlated with nurse role; however, we found that with adjustment for nurse role and perceived preparedness, nurse prescribers had greater probability of within-practice follow-up than non-prescribers. This is counterintuitive because it is reasonable to surmise that practice nurses who could prescribe would be less likely to refer because they did not need to refer the patient onto a prescribing clinician. However, as there were only three practice nurses with the ability to prescribe this finding should be viewed with caution. The other analyses we carried out found no evidence to indicate that possession of an undergraduate nursing degree; prior telephone triage experience, or experience in a wider variety of clinical settings, were associated with recommendation for within-practice follow-up.

### 6.1 Strengths and limitations

Strengths of the study include the large number of patients in the sample taken from 14 practices in four areas of the U.K. It is therefore likely that our findings are generalisable to the population of patients seeking same-day consultations in primary care. All nurses received the same training and were therefore broadly comparable across practices for how they delivered telephone triage using CDSS within the context of the ESTEEM trial. This analysis adds to the previously limited evidence on nurse telephone triage in routine in-hours primary care.

This study provides useful data on variation in nurse triage call disposal but limitations must be acknowledged. Firstly, only 35 nurses out of the 45 who carried out nurse triage as part of the trial were included in the analysis. Given the small number of nurses, and the fact that seven nurse characteristics were included in the analyses, a degree of correlation across characteristics was inevitable. We have taken the pragmatic approach of including all characteristics in the analyses, as we had no a priori hypotheses regarding how the characteristics may have been correlated until we were able to view the data. However, we acknowledge the possibility of spurious results due to correlation between variables, or to small numbers of nurses with specific combinations of characteristics. A larger study with a greater number of nurses would help mitigate these issues. We also acknowledge that whilst all nurses had standardised training, they did not have a long time to consolidate their skills in triage during the trial therefore the results may not be generalisable to nurses who are given a longer period to consolidate their practice and gain a wider experience of triage. Our analysis focuses on understanding the frequency and distribution of patient calls and in-practice follow-up arising from nurse triage. A limitation of this approach is that we can make no judgements regarding the quality of care. Specifically we cannot explore the appropriateness of the triage decisions made, or how much variation in outcomes was due to differential use of CDSS.

### 6.2 Implications for practice

We identified various determinants of differences in nurse telephone triage call dispositions. While the majority of patients seeking a same-day appointment request were recommended for follow-up within the practice by triaging nurses (86%), the proportion of patients referred for follow-up varied significantly between individual nurses. Thus an understanding of the factors influencing the decision-making processes would be helpful for practices in determining the qualities that may be desirable in selecting a nurse to perform triage. If all nurses could achieve a proportion of patients managed by self-care of approximately 65% (the lowest proportion in this study by an individual nurse was 63%), this would represent a clinically significant reduction in workload on the day of the initial patient request (assuming that referral to self-care was made appropriately and that the patient did not need to return to the practice to seek further advice with the same problem, or did not seek medical assistance elsewhere).

We have demonstrated that perceived preparedness and job role are potentially important factors to bear in mind when considering introducing telephone triage to a nurse's role. Other research suggests that training could have a beneficial impact on triage outcomes (Giesen et al., 2007, Moll van Charante et al., 2006). Our study indicates this may be necessary, but not sufficient, as all of the nurses had received the same level of training, yet the perceived preparedness for triage still varied. Nurses come to the training with different backgrounds, personalities, skills and experience. CDSS mediated telephone triage to manage acute symptoms requires a reconfiguration of skills from traditional nurse roles of face-to-face patient encounters, particularly chronic illness reviews which involve ongoing symptoms and nurse–patient relationships built over time. Nurses will therefore respond to telephone triage training in a range of ways which all may contribute to how they report perceived preparedness. This generates an area of further research to develop a training programme that takes a more nuanced consideration of what influences a nurse's perceived preparedness to carrying out telephone triage and the resulting impact of this training on triage outcomes.

## 7 Conclusion

We observed important differences in patterns of call management between nurses who carried out nurse telephone triage. Our triage intervention included standardised training delivered to nurses with, for the most part, considerable primary care experience. Despite this, nursing characteristics independently influenced how telephone triage was implemented. Nurse practitioners, and nurses who reported feeling ‘well-prepared’ for this role, were more likely to manage the call definitively without recommending further follow-up compared with their counterparts. Practices considering implementing nurse telephone triage to manage same-day appointment requests should ensure that nurses transitioning into their new roles feel adequately prepared; adapting telephone triage training packages towards individual nurse skills and backgrounds.

## Figures and Tables

**Table 1:** Self-reported characteristics of nurses.

Nurse characteristic	Total sample (*N* = 35)
Gender *n* (%)	
Female	35 (100%)
Male	0 (0%)
Ethnicity *n* (%)	
White	34 (97%)
Other	1 (3%)
Age (years) *n* (%)	
18–24	0 (0%)
25–34	1 (3%)
35–44	11 (31%)
45–54	19 (54%)
55–64	4 (11%)
Self-reported job role *n* (%)	
Nurse practitioner	8 (22%)
Practice nurse	27 (77%)
No. of years clinical experience (years) *n* (%)	
0–9	4 (11%)
10–19	7 (20%)
20 or more	24 (69%)
Ability to prescribe *n* (%)	
Yes	11 (31%)
No	24 (69%)
Education (highest qualification) *n* (%)	
Below degree level	23 (66%)
Degree or above	12 (34%)
Prior experience of telephone triage in *n* (%)	
General practice	5 (14%)
Primary care	3 (9%)
A & E^a^	4 (11%)
OOHs^b^	3 (9%)
NHS Direct	0 (0%)
Not indicated	1 (3%)
Prior experience of CDSS *n* (%)	
Yes	2 (6%)
No	31 (89%)
No response	2 (6%)
How well prepared they felt for triage *n* (%)	
Very well prepared	18 (51%)
Moderately well	11 (31%)
Not well	4 (11%)

a Accident and emergency.

b Out of hours service.

**Table 2:** Multivariable hierarchical logistic regression models including individual nurse characteristics, and all nurse characteristics combined, with regard to within practice follow-up.

Nurse characteristic(s)	Odds ratio for within practice follow-up (95% CI)	Global *p*-value	Number of nurses (between nurse SD)	Number of patients
Models with one nurse characteristics^a^				
Nurse practitioner status (reference: practice nurse)				
Nurse practitioner	0.45 (0.24–0.82)	0.010	33 (0.68)	4141
Prescriber status (reference: non-prescriber)				
Prescriber	0.59 (0.33–1.05)	0.073	35 (0.69)	4433
Years’ nursing experience (reference; 20 years or more)				
10–19 years	1.29 (0.66–2.52)	0.401	35 (0.71)	4433
0–9 years	1.96 (0.68–5.68)			
Degree qualification (reference: no)				
Yes	0.92 (0.49–1.74)	0.793	35 (0.74)	4433
Triage experience (reference: no)				
Yes	0.37 (0.22–0.63)	<0.001	35 (0.58)	4433
Number of settings of nurse experience (reference: 1 or 2)				
3 or more	0.58 (0.31–1.06)	0.074	35 (0.69)	4433
Perceived preparedness (reference: well-prepared)				
Not well/moderately well	2.89 (1.65–5.07)	<0.001	33 (0.58)	4166

Model with all seven nurse characteristics^a^				
Practice nurse	0.20 (0.08–0.50)	0.001	31 (0.40)	3874
Prescriber	2.00 (0.81–4.98)	0.135		
Years’ nursing experience (reference; 20 years or more)				
10–19 years	0.74 (0.41–1.35)	0.591		
0–9 years	0.65 (0.20–2.11)			
Degree qualification	1.60 (0.91–2.82)	0.102		
Triage experience	0.83 (0.47–1.49)	0.539		
3 or more settings of nurse experience	0.71 (0.44–1.16)	0.173		
Not well/moderately well prepared	2.32 (1.18–4.54)	0.015		

CI, confidence interval; SD, standard deviation.

a All models adjust for patient- and practice-level characteristics.

**Table 3:** Multivariable hierarchical logistic regression model including nurse characteristics significantly associated with within practice follow-up.

Nurse characteristic	Odds ratio (95% CI) for within practice follow-up^a^^,^^b^^,^^c^	*p*-Value	Marginal probability (95% CI) for within practice follow-up
Nursing role			
Practice nurse (referent)	–		0.90 (0.88–0.93)
Nurse practitioner	0.19 (0.07–0.49)	0.001	0.66 (0.51–0.81)
Preparedness for triage			
Very well (referent)	–		0.57 (0.40–0.75)
Not well/moderately well	3.17 (1.81–5.55)	<0.001	0.79 (0.74–0.85)
Prescriber status			
Non-prescriber (referent)	–		0.77 (0.69–0.86)
Prescriber	3.15 (1.21–8.16)	0.018	0.90 (0.87–0.94)

CI, confidence interval.

a Analysis adjusted for patient- and practice-level characteristics.

b Analysis included 3874 patients managed by 31 nurses.

c Between nurse standard deviation: 0.48.
